# Multi-Omics Classification of Intrahepatic Cholangiocarcinoma: A Systematic Review and Meta-Analysis

**DOI:** 10.3390/cancers16142596

**Published:** 2024-07-20

**Authors:** Laura Alaimo, Sara Boggio, Giovanni Catalano, Giuseppe Calderone, Edoardo Poletto, Mario De Bellis, Tommaso Campagnaro, Corrado Pedrazzani, Simone Conci, Andrea Ruzzenente

**Affiliations:** Department of Surgery, Dentistry, Gynecology, and Pediatrics, Division of General and Hepato-Biliary Surgery, University of Verona, University Hospital G.B. Rossi, 37134 Verona, Italy; laura.alaimo@univr.it (L.A.);

**Keywords:** multi-omics, intrahepatic cholangiocarcinoma, bile duct cancer, classification, molecular signature

## Abstract

**Simple Summary:**

The prognosis of intrahepatic cholangiocarcinoma (ICC) depends on tumor biology, morphology, and the high rates of late diagnosis and recurrence after curative-intent surgery, with high heterogeneity. Understanding the molecular landscape of ICC and its clinical implications for treatment and prognosis remains a major challenge. Several attempts have been made to classify molecular ICC subtypes, but they have produced heterogeneous results and need a clear clinical definition. This systematic review examined the evidence for the multi-omics-based classification of ICC to assess its clinical and prognostic significance. Molecular analysis of ICC can assist in decision-making by identifying patients who are suitable for targeted pre- and postoperative chemotherapy. Identifying clinical characteristics associated with specific ICC clusters resulting from multi-omics analysis could help tailor patient management. However, there are logistical, technical, and therapeutic challenges in the routine application of ICC molecular characterization. Identifying clinical characteristics commonly found in specific ICC clusters can help define high-risk ICC subtypes quickly and assist in selecting patients who may benefit the most from molecular profiling in the disease’s earlier stages.

**Abstract:**

Intrahepatic cholangiocarcinoma (ICC) is a heterogeneous disease characterized by a dismal prognosis. Various attempts have been made to classify ICC subtypes with varying prognoses, but a consensus has yet to be reached. This systematic review aims to gather relevant data on the multi-omics-based ICC classification. The PubMed, Embase, and Cochrane databases were searched for terms related to ICC and multi-omics analysis. Studies that identified multi-omics-derived ICC subtypes and investigated clinicopathological predictors of long-term outcomes were included. Nine studies, which included 910 patients, were considered eligible. Mean 3- and 5-year overall survival were 25.7% and 19.6%, respectively, for the multi-omics subtypes related to poor prognosis, while they were 70.2% and 63.3%, respectively, for the subtypes linked to a better prognosis. Several negative prognostic factors were identified, such as genes’ expression profile promoting inflammation, mutations in the KRAS gene, advanced tumor stage, and elevated levels of oncological markers. The subtype with worse clinicopathological characteristics was associated with worse survival (Ref.: good prognosis subtype; pooled hazard ratio 2.06, 95%CI 1.67–2.53). Several attempts have been made to classify molecular ICC subtypes, but they have yielded heterogeneous results and need a clear clinical definition. More efforts are required to build a comprehensive classification system that includes both molecular and clinical characteristics before implementation in clinical practice to facilitate decision-making and select patients who may benefit the most from comprehensive molecular profiling in the disease’s earlier stages.

## 1. Introduction

Cholangiocarcinoma (CCA) is a biliary tract cancer with high levels of phenotypical and clinical heterogeneity. According to the World Health Organization (WHO), this malignancy can be classified as intrahepatic (ICC) or extrahepatic (ECC) based on its anatomical site of origin [1]. ICC is the second most common primary liver cancer after hepatocellular carcinoma and is becoming more prevalent worldwide [2]. However, ICC has a poor prognosis due to the high rate of late diagnosis, aggressive nature, and chemotherapy resistance [3]. This is mainly due to its insidious onset, which makes it asymptomatic in the early stages, resulting in a late diagnosis [4]. Additionally, the available treatment options are limited. Surgical resection is the only curative-intent treatment [5,6]. However, early diagnosis, precise staging, and personalized and multidisciplinary management are challenging due to the high heterogeneity of ICC at clinical, genomic, epigenetic, and molecular levels [7,8]. Identifying clinical, histological, and molecular characteristics that define different subtypes of ICC with clinical implications for treatment and prognosis remains a major challenge.

According to the EASL-ILCA Clinical Practice Guidelines on the management of ICC [9], the histological subclassification of ICC into large and small duct types is suggested due to their different clinicopathological features and mutation profiles with subsequent changes in patient management based on their prognostic and therapeutic implications. Specifically, compared with the large duct type, the small duct type is characterized by better long-term outcomes with less aggressive pathological features (i.e., lymphatic and/or perineural invasion) and more frequent genetic alterations (i.e., IDH1/2 or BAP1 mutations, and FGFR2 fusions), which are treatable with promising targeted therapies. For these reasons, recognizing the different ICC subtypes and utilizing targeted therapies can significantly enhance the management of cholangiocarcinoma.

In recent years, advanced molecular profiling platforms have provided invaluable insights into cancer biology. These findings revealed that alterations in gene expression can play a critical role in the progression, development, and heterogeneity of CCA [10,11]. Moreover, molecular profiling has helped identify new potential actionable therapeutic targets. Specifically, multi-omics studies have uncovered a complex and heterogeneous landscape of CCA, revealing distinct molecular subtypes and commonly shared mutations [12]. Based on these characteristics and considering the limited effectiveness of second-line chemotherapy, the European Society for Medical Oncology (ESMO) guidelines recommend Next Generation Sequencing (NGS) for all patients with CCA and propose the ESCAT (Scale for Clinical Actionability of Molecular Targets) classification. Specifically, mutations associated with CCA are classified as ready for routine use (i.e., IDH1 mutations, FGFR2 fusions, high microsatellite instability, and NTRK fusions), undergoing experimentation due to the unknown extent of the benefit (i.e., BRAF V600E mutations), and hypothetical target due to clinical studies in other tumor types or similar molecular alterations (i.e., HER2 alterations) [9].

Although several multi-omics studies have attempted to characterize and categorize the ICC subtypes widely, they reported heterogeneous results that still need to be compared and unified and need a clear clinical definition, which limits their applicability. Identifying clinical characteristics associated with specific ICC molecular clusters resulting from the multi-omics analysis could help tailor patient management, leading to more effective and timely decision-making. Additionally, routine ICC molecular characterization poses logistical, technical, and therapeutic challenges. As such, identifying clinical characteristics commonly found in specific ICC clusters can help define high-risk ICC subtypes quickly and assist in selecting patients who may benefit most from molecular profiling in the disease’s earlier stages. The association between molecular patterns and clinicopathological characteristics represents a critical factor in enhancing the comprehension of the disease and developing precise diagnostic and therapeutic strategies.

This systematic review and meta-analysis sought to collect the relevant available data regarding multi-omics-based profiles of ICC to find any associations between molecular, clinical, and prognostic characteristics. In addition, the heterogeneity among the subtypes that are currently classified in the literature, as well as the knowledge consensus or gaps, were investigated.

## 2. Materials and Methods

### 2.1. Study Selection

**Figure 1:**
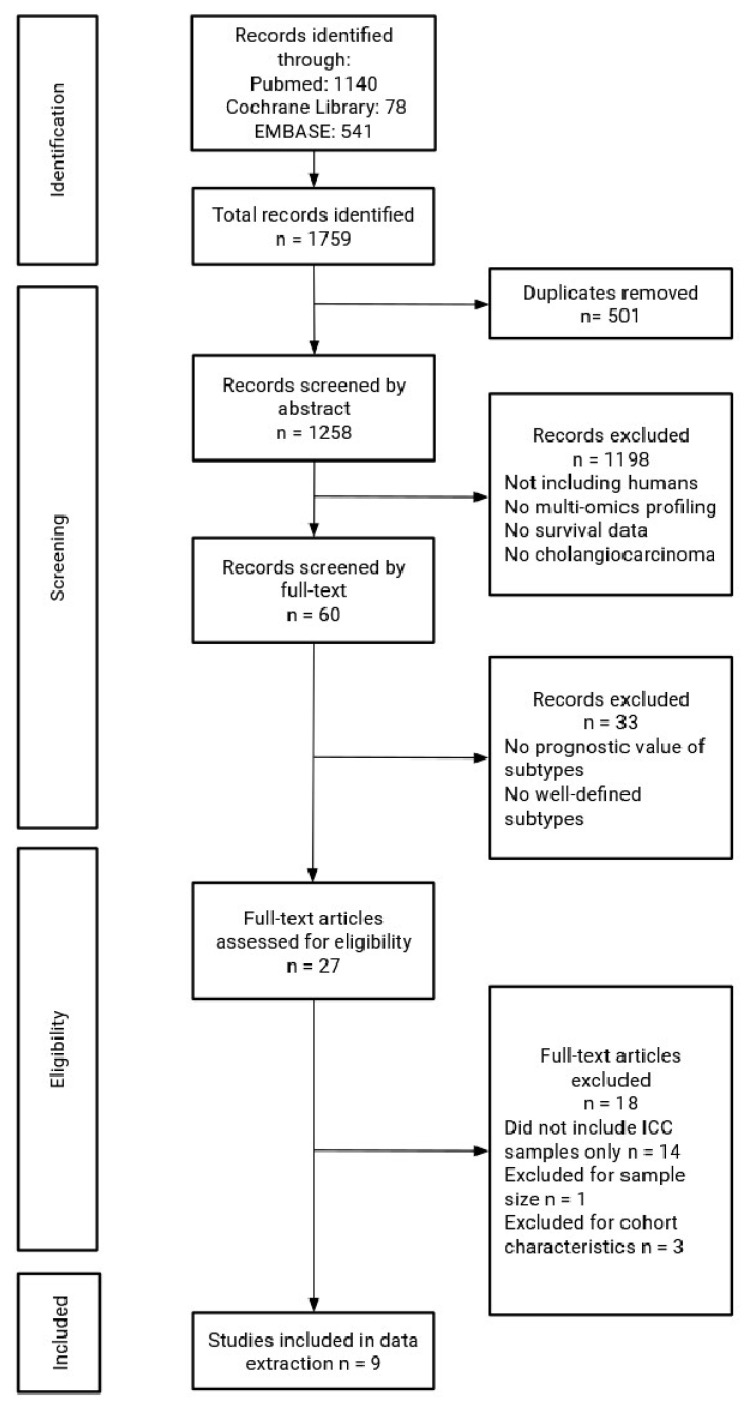
Flow diagram of study selection.

A literature search for relevant studies was conducted in June 2024 using the PubMed, Cochrane, and Embase databases. The search strategy was established following consensus among the authors. The following search terms were employed: “cholangiocarcinoma” AND “molecular” AND “subtype”, “cholangiocarcinoma” AND “mutations” AND “subtype”, “cholangiocarcinoma” AND “prognostic” AND “molecular” AND “groups”, “cholangiocarcinoma” AND “molecular”, “cholangiocarcinoma” AND “transcriptomics”, “cholangiocarcinoma” AND “molecular” AND “groups”, “cholangiocarcinoma” AND “molecular” AND “clinical”, “cholangiocarcinoma” AND “prognosis” AND “clinical”, “cholangiocarcinoma” AND “prognosis” AND “omics”; the term “review” was always excluded through the NOT function. Only studies published in the English language were considered eligible. Eligible studies were collected, and additional relevant publications were retrieved through cross-referencing. Eligible studies addressed multi-omics-derived gene expression signatures of CCA samples that were obtained from patient specimens. Only studies that investigated the correlation between the ICC molecular subtypes and prognosis were eventually included (Figure 1). This study was conducted by following the Preferred Reporting Items for Systematic Reviews and Meta-Analyses (PRISMA) and in accordance with the Declaration of Helsinki [13]. The study protocol was not registered. No new data were created or analyzed in this study. Data sharing is not applicable to this article.

The outcome of interest was the definition of well-characterized ICC subtypes with specific gene expression signatures. Any potential associations between the ICC subtypes, clinical characteristics, and prognosis were assessed. All articles that were found using the formal search strategy were imported into CADIMA (version 2.2.4.2, Julius Kühn-Institut, Quedlinburg, Germany) [14]. CADIMA is a free web tool that facilitates the conduct and assurance of systematic review documentation. This tool allows for automated duplicate removal and ensures a computerized allocation of records during the screening process (considering the degree of a potential independent and parallel assessment). Multiple authors screened the studies in parallel (SB, GC, LA, GC). Specifically, four investigators independently performed the title and abstract review (SB, GC, LA, GC). Two investigators independently retrieved the full texts of eligible articles and assessed the inclusion criteria (SB, LA). Inconsistencies and disagreements were resolved by consensus among the authors. The data extraction process was limited to studies on ICC.

### 2.2. Data Extraction and Quality Assessment

Two reviewers (SB and GC) independently performed data extraction. Extracted data included the study characteristics (i.e., first author, journal and year of publication, study design, number of patients, and validation dataset). Main data categories were clinical data such as patient preoperative characteristics (i.e., age and ethnicity), disease characteristics (i.e., viral hepatitis, cirrhosis, carcinoembryonic antigen [CEA] levels, carbohydrate antigen 19-9 [CA19-9] levels), tumor characteristics (i.e., TNM stage according to the American Joint Committee on Cancer [15], histological type (small duct or large duct), size and number of lesions, vascular [VI] and perineural invasion [PNI]), and long-term outcomes (i.e., overall [OS] and disease-free survival [DFS]). Molecular and technical data such as sequencing platform, multi-omics analysis, clustering method, the number of clusters, and the subtypes’ most common mutational and gene expression characteristics were collected. If the studies did not provide complete data on long-term outcomes, Engauge Digitizer 11.1 software was utilized to extract the survival rate at the corresponding time point from the survival curves reported by the study (http://plotdigitizer.sourceforge.net, accessed on 23 November 2023).

Two reviewers (SB and LA) performed a quality assessment of the studies following the Newcastle–Ottawa Quality Assessment Scale. Specifically, 1 point was assigned for each of the following parameters: adequate patient selection and sample size, measurement of molecular markers, strength and suitability of clustering method, outcome assessment such as gene signature, correlation to prognosis, and collection of clinicopathological characteristics.

### 2.3. Statistical Analysis

For descriptive statistics, categorical variables were reported as counts and percentages (%). Continuous variables were summarized as median (interquartile range [IQR]) or mean (standard deviation [SD]), as appropriate. Publication bias was assessed using the Egger test by the funnel plot method. All studies used the Kaplan–Meier method, log-rank test, and Cox regression analysis to estimate and compare OS and DFS between clusters. Clusters with better OS rates were defined as the “Good prognosis” subtype, and those with worse OS rates were defined as the “Bad prognosis” subtype. The generic inverse variance method for meta-analysis was used for survival data pooling with the DerSimonian–Laird estimator to estimate the between-study variance. A random effects meta-analysis model was used. Pooled hazard ratios (HRs) with 95% confidence intervals (95% CI) were calculated using each study’s log(HR) and its standard error (SE) log(HR). The results were depicted using forest plots. The mathematical characteristics of logarithmic transformations and how they affect values on various scales and ranges are responsible for the slight variations in the transformed HR values reported in the forest plots compared to the ones reported by the studies. However, the process of transformation and back-transformation maintains the overall pattern and interpretation of the HR ratios [16,17]. Review Manager (RevMan, version 5.2 software Metagen package) was used for forest plots. Statistical heterogeneity between studies was assessed, with I^2^ > 50% indicating significant heterogeneity. The statistical analyses were conducted utilizing R version 4.2.0 (R Project for Statistical Computing).

## 3. Results

### 3.1. Search Results

Among 1759 articles imported into CADIMA, 13 studies fulfilled the inclusion criteria. However, two studies were excluded at this stage due to the review design, the small sample size (<20 patients) [18], and the use of previously published results [19]. Two studies that analyzed merged data from previously published cohorts, and publicly available datasets were also excluded [12,20]. As a result, nine studies that analyzed a unique cohort of patients with ICC were selected, and data extraction was performed (Figure 1). All studies reported data on OS, while four reported data on both OS and DFS [21,22,23,24]. The results of the quality assessment of the studies are reported in Table S1.

Overall, the studies included 910 patients with a mean age of 62.1 years (Range 59.5–65.0) (Table 1). All patients had a confirmed diagnosis of ICC. Five out of nine studies were Eastern (*n* = 527 patients, 57.9%) [21,24,25,26,27], while the remaining studies were Western (*n* = 383 patients, 42.1%) [22,23,28,29].

### 3.2. Multi-Omics Classification and Cluster Analysis

The review included studies that used multi-omics analyses to define ICC clusters, addressing their correlation with long-term outcomes. Five studies used transcriptomic sequencing [21,22,23,27,29], three studies used proteomic sequencing [24,25,26], and one study used integrated multi-omics platforms to identify ICC distinctive clusters [28]. Multi-profiles were generated by gene expression analysis through RNA sequencing or microarrays, proteomic analysis, copy number alterations (CNAs), and exome and methylome sequencing, demonstrating an intricate ICC molecular landscape.

Cluster analyses were employed based on their suitability for the studies’ aim and sample size. Eight studies (88.9%) [21,22,24,25,26,27,29,30] used hierarchical clustering, while one study (11.1%) [28] utilized integrative cluster analysis to integrate several data from various sequencing platforms (Table S2). After the clustering, some studies performed genomic and transcriptomic analyses to further characterize the identified subtypes [24,25,26].

The number of clusters identified by the studies ranged from 2 to 4 (Table 2). Each study identified at least one cluster with a better prognosis (i.e., “Good prognosis cluster”) and one with the worst prognosis (i.e., “Bad prognosis cluster”). Only one study did not validate the cluster analysis results in a separate cohort [28], while the other studies found concordance between the discovery and validation cohorts [21,22,23,24,25,26,27,29]. Six studies (66.6%) [21,22,23,24,27,29] proposed a gene signature, often referred to by the term “classifier” or “survival signature”, which was further validated in an external cohort. Specifically, the Andersen et al. [22] classifier was highly concordant (*p* < 0.001) with the Oishi et al. [27] classifier but moderately concordant (*p* < 0.05) with the Sia et al. [23] classifier [29].

Table 3 provides an overview of the most important molecular characteristics, markers, and prognosis associated with each cluster. The mutation rates of KRAS, TP53, FGFR2, and IDH1 ranged from 8.0% to 24.6% [21,22,23,25,26,28], 9.7% to 21.0% [21,25,26,28], 0 to 13.0% [21,26], 10.2% to 17.0% [21,26,28,29], respectively. Overall, mutations in KRAS and TP53 were associated with poor prognosis [21,22,23,24,26,29], while FGFR2 alterations and IDH1 mutation were associated with a better prognosis [21,23,24,25,26]. Only Andersen et al. [22] reported higher rates of BRAF mutation among patients from the bad prognosis ICC subtype, and only Bao et al. [25] reported higher rates of ARID1A mutation among patients from the good prognosis ICC subtype. Other mutations, such as BAP1 and ERBB2, have been variably reported by the studies (Table S3). However, these mutations were not specifically associated with any ICC cluster.

Gene set enrichment analysis (GSEA) and ingenuity pathway analysis (IPA) were the most commonly used analyses for examining pathway enrichment associated with upregulated/downregulated genes among subgroups. Notably, an unfavorable prognosis was related to the enrichment of inflammatory, mesenchymal, or proliferation genes and pathways [21,22,26,27,29]. However, Oishi et al. [27] found that a bad prognosis was linked with the mesenchymal subtype only when it had more stem-cell-like features. A better OS was associated with an enrichment of immune-related pathways [22,29].

### 3.3. Long-Term Outcomes

Overall, among patients in the good prognosis clusters, the 5-year OS ranged from 39.8% to 78.0% (mean, 63.3%). In contrast, patients in the bad prognosis clusters had a 5-year OS ranging from 7.6% to 30.0% (mean, 19.6%) (Table 4). Patients in the good prognosis clusters had a 5-year DFS ranging from 4.6% to 47.0% (mean, 28.6%), while among patients belonging to the bad prognosis clusters, the 5-year DFS ranged from 0% to 22.3% (mean, 16.2%) [21,22,23,24].

**Figure 2:**
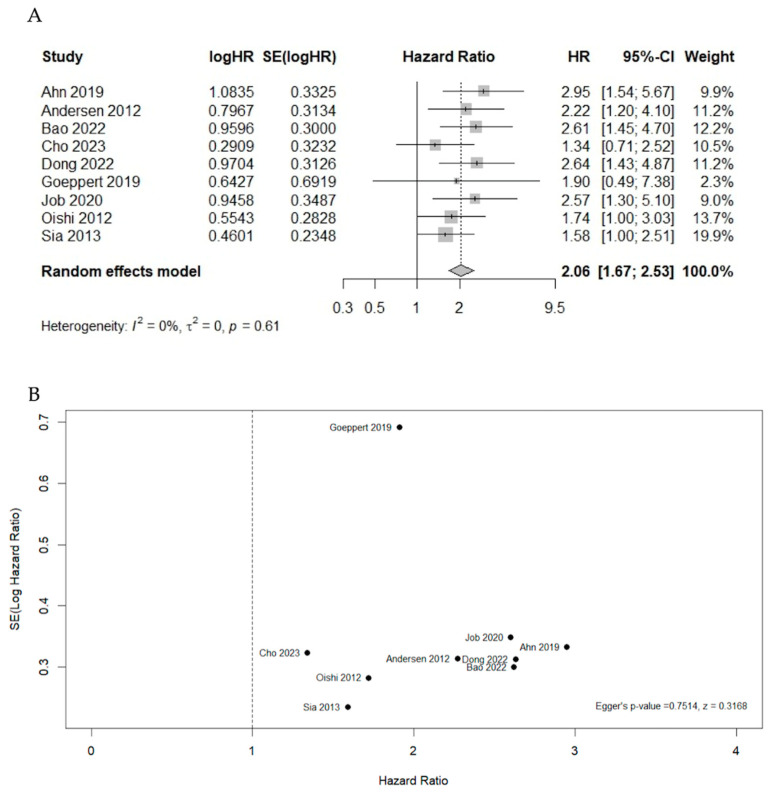
Forest plot for overall survival based on univariable analysis: (**A**) all studies; (**B**) funnel plot for publication bias [21,22,23,24,25,26,27,28,29].

Pooled HR for overall survival among bad prognosis clusters was 2.06 (95%CI 1.67–2.53) (I^2^ = 0%, *p* > 0.61) (Figure 2A, Table S4). The funnel plot of the studies is presented in Figure 2B, with the Egger test indicating the absence of significant publication bias (intercept = 0.7514, *p* = 0.3168) [21,22,23,24,27,28,29]. Figure S1 depicts the forest plot for OS based on multivariable analyses; however, the data were limited.

### 3.4. Cluster-Specific Clinical and Prognostic Characteristics

Patient and tumor characteristics are summarized in Table S5. Only two studies (22.3%) [26,28] reported the mean CEA levels (2.7–2.9 μg/L), and three studies (37.5%) [26,28,29] reported mean CA19-9 levels (46.2–83.3 U/mL). Four studies (50.0%) [21,23,26,28] reported the mean tumor size (5.5–7.2 cm), and four studies (50.0%) [21,25,26,28] reported the TNM stage, with a majority of patients having stage I-II disease (I, 39.5% [30.8–50.0%]; II, 30.2% [16.3–53.8%]). Four studies reported the rates of PNI (mean, 35.7% [17.0–76.9%]) [23,26,28,29] and five the rates of VI (mean, 40.7% [13.0–65.4%]) [21,23,24,26,29].

The TNM stage III–IV (pooled HR 2.75 [95%CI 1.83–4.11]; I^2^ = 0%, *p* = 0.56) and the presence of PNI (pooled HR 1.91 [95%CI 1.17–3.14]; I^2^ = 31%, *p* = 0.23) or VI (pooled HR 1.89 [95%CI 1.32–2.72]; I^2^ = 0%, *p* = 0.44) had a negative impact on patients’ OS with no heterogeneity among the studies (Table S6, Figure S2). The funnel plot of the studies is presented in Figure S2D, with the Egger test indicating the absence of significant publication bias (intercept = −0.3258, *p* = 0.97).

Some adverse clinical characteristics were distributed differently between the good and bad prognosis clusters (Table 5), with the bad prognosis cluster having higher levels of CEA [21,26], higher rates of HBV infection [21], nodal metastases [26], histological large duct type [21], PNI [23,25,27], and a more advanced TNM stage [26]. However, only three studies (37.5%) [21,26,29] thoroughly evaluated the potential association between these clinicopathological characteristics and the molecular clusters of ICC.

## 4. Discussion

Intrahepatic cholangiocarcinoma (ICC) is a rare disease with a dismal prognosis. In recent years, an increase in its incidence and prevalence has been reported. Surgery is the primary treatment option, but the recurrence rates after curative-intent resection are still high [30,31,32]. This highly heterogeneous malignancy is mainly classified based on tumor location and histological characteristics, as a standardized molecular classification has not yet been established [33]. Identifying distinct tumor subgroups is crucial for tailoring ICC management and treatment and ensuring optimal outcomes following curative-intent resection using targeted therapy and immunotherapy [34]. Recognizing the molecular profile and utilizing targeted therapies can significantly enhance the management of cholangiocarcinoma. However, the routine application of ICC molecular characterization presents logistical, technical, and therapeutic challenges that cannot be overlooked. In this regard, identifying clinical characteristics that are commonly found in specific ICC clusters can help quickly identify high-risk ICC subtypes. This can also assist in selecting patients who may benefit the most from early molecular profiling using a comprehensive panel in the disease’s earlier stages. In recent years, multi-omics studies have revealed a complex and diverse landscape of ICC, resulting in the identification of distinct molecular subtypes [11,35,36,37]. Despite being characterized by different studies, the results are heterogeneous and have yet to be compared and unified. In addition, few studies have investigated the clinical factors associated with molecular subtypes of ICC, with most of the studies reporting only data on long-term outcomes and few clinical characteristics. As such, the need for a comprehensive and unique clinicopathological and molecular definition of ICC subtypes limits the clinical applicability of molecular classification systems.

This review is important as it clarifies the evidence of molecular-based ICC subtype classification with prognostic value and correlation with clinical factors. The review emphasizes the need to link molecular characteristics with clinical factors before integrating ICC molecular assessment and classification into clinical practice. This knowledge is essential for identifying clinical markers of ICC clusters with different prognoses, selecting patients who should undergo tumor molecular characterization, identifying targeted therapies, and tailoring patient management promptly.

In this systematic review, we comprehensively analyzed the available data from multi-omics sequencing (transcriptomic, proteomic, and integrated multi-omics) to identify distinctive ICC clusters with various prognoses. Only studies that found an association between ICC multi-omics clusters and long-term outcomes were included. The studies consistently indicated two to four molecular clusters of ICC, each showing a significant association with survival despite variations in multi-omics platform, sample size, number of subtypes detected, and clustering technique. The choice between various clustering techniques in multi-omics analysis usually depends on specific goals, available data, and prior knowledge. Most studies utilized an unsupervised clustering method, which helps examine the underlying structure of gene expression data without any predetermined groupings or labeled variables. However, some expectations regarding the number of clusters may be present [38]. As such, this approach is widely used nowadays due to its effectiveness in identifying markers and classifying modules. On the other hand, the integrative clustering method is used in studies that obtain clusters from data deriving from multiple sequencing platforms, allowing for a more comprehensive understanding of relationships between biological systems [28]. Out of nine proposed studies, 6 (66.6%) [21,22,23,24,27,29] developed a gene signature referred to as a “classifier” or “survival signature”. This signature was validated externally and differentiated individuals in outcome-linked subtypes, demonstrating prognostic value. Additionally, Job et al. [29] addressed the concordance between classifiers from different publications. They found that Andersen et al. [22] classifier was highly concordant (*p* < 0.001) with Oishi et al. [27] classifier but moderately concordant (*p* < 0.05) with Sia et al. [23] classifier.

According to the EASL-ILCA Clinical Practice Guidelines on the management of ICC [9], the histological subclassification of ICC into large and small duct types is suggested due to their different clinicopathological features and mutation profiles with subsequent clinical utility based on their prognostic and therapeutic implications. Specifically, compared with the large duct type, the small duct type is characterized by better long-term outcomes with less aggressive pathological features, such as lymphatic and/or perineural invasion, and more frequent genetic alterations, such as IDH1/2 or BAP1 mutations, and FGFR2 fusions, which are treatable with currently available and promising targeted therapies, such as Pemigatinib for ICC with FGFR2 fusion/rearrangement or Ivosidenib for ICC with IDH1 mutations. On the other hand, the large duct type often presents with KRAS and SMAD4 mutations. In line with the current literature, three studies reported that the large duct ICC was more frequent among patients belonging to the “bad prognosis” cluster [21,22,28]. These findings confirm that recognizing the molecular profile and utilizing targeted therapies can significantly enhance ICC management and prognosis.

In the studies analyzed, at least two molecular subtypes were identified and characterized by survival analysis. This information allowed for the classification of clusters into good prognosis and bad prognosis ICC subtypes. Notably, patients in the good prognosis cluster had a mean 3-year OS of 70.2%, while 3-year OS was only 25.7% in the bad prognosis cluster. A previous international multicentric study reported a median 3-year OS of 61.4% in the good prognosis cluster versus 47.3% in the bad prognosis cluster [39]. In this study, Alaimo et al. used tumor burden score (TBS), CA19-9 levels, and neutrophil-to-lymphocyte ratio (NLR) to create a phenotype-based ICC classification. Using unsupervised cluster analysis, they defined three types of ICCs (i.e., common, proliferative, and inflammatory); however, molecular and gene expression data were unavailable. This emphasizes the need for a comprehensive characterization of ICC subtypes encompassing both clinical and molecular features. Notably, in the current review, the multi-omics subtype classification strongly influenced the OS (Ref.: good prognosis cluster; bad prognosis cluster: HR 2.06 (95%CI 1.67–2.53) (I^2^ = 0%, *p* < 0.61).

Based on clinical evidence of the benefits of inhibitors targeting specific genetic abnormalities in certain groups of patients with advanced cholangiocarcinoma, and considering the limited effectiveness of second-line chemotherapy, the European Society for Medical Oncology (ESMO) guidelines recommend NGS for all patients with cholangiocarcinoma and propose the ESCAT (Scale for Clinical Actionability of Molecular Targets) classification [9]. Specifically, mutations associated with cholangiocarcinoma are classified as ready for routine use (i.e., IDH1 mutations, FGFR2 fusions, high microsatellite instability, and NTRK fusions), undergoing experimentation due to the unknown extent of the benefit (i.e., BRAF V600E mutations), and hypothetical target due to clinical studies in other tumor types or similar molecular alterations (i.e., HER2 alterations). According to current literature, KRAS mutation is the most common alteration in patients with ICC, observed in 8.0–50.0% of cases [37,40]. In line with previous studies, the current review reported KRAS mutation in 8.0–24.6% of cases. Dong et al. [26] and Andersen et al. [22] found a significant association with OS, with KRAS mutation being a negative prognostic factor. Among the included studies, TP53 mutation was reported in 9.7–21.0% and was associated with worse OS. IDH1 mutations are commonly found in CCA, comprising 20.0–30.0% of cases, mainly intrahepatic. However, the association between IDH mutation and bad or good prognosis is unclear [37,41,42,43]. In the current review, the reported rate of IDH1 was 10.2–17.0%, making it the second most common alteration. Two studies reported the rate of FGFR2 mutations and fusion products (0–13.0%) [21,26]. In the literature, FGFR2 alterations are highly actionable and have been reported in 6.0–17.0% of ICC cases, being more common among Caucasian and Asian patients [44,45,46]. These findings highlight that specific subgroups of patients with ICC could benefit from targeted therapy. However, being a small proportion, the definition of clinical characteristics that are commonly found in patients with targetable mutations can help select patients who may benefit the most from early molecular profiling. These patients should be promptly referred to highly specialized centers that can provide the best care due to the availability of specific clinical trials.

Additionally, most studies conducted molecular analysis and identified prognostic subgroups with common features relative to gene expression and mutational characteristics. Inflammation has been linked to the development of ICC and metastases by modulating tumor microenvironment (TME) components [47]. In line with the literature, the enrichment of inflammation-related genes and pathways was mainly linked to poorer outcomes, with lower survival times and rates [21,25,26]. Bao et al. [25] utilized a clustering technique based on genes associated with the TME and an extra level of single-cell analysis to observe the correlation between inflammation and more aggressive phenotypes. Mesenchymal tissue contains high concentrations of paracrine and vascular factors produced by fibroblasts, which the immune system activates. These factors may promote pro-tumorigenic pathways and hinder the recruitment of immune cells [48]. Genes and pathways involved in mesenchymal transformation and proliferation have been associated with poor prognosis. Furthermore, these factors play a critical role in producing a fibrous stroma. ICC typically displays a substantial desmoplastic stroma that comprises a blend of many non-immune and immune cell types. These include cancer-associated fibroblasts (CAFs) and tumor-associated macrophages; both considered negative prognostic factors [49]. Sia et al. [23] and Goeppert et al. [28] found that mesenchymal, proliferative, and angiogenic pathways and genes are upregulated and associated with poor prognoses. However, according to Oishi et al. [27], this specific subtype of ICC was associated with a poor prognosis only when it had more stem-cell-like features. This could be due to the promotion of the development of cancer stem cells (CSCs), which are generated through the epithelial-to-mesenchymal transition (EMT) [48]. Job et al. [29] found that an ambivalent immune profile, composed of immune-stimulating and immune-suppressive factors, is related to better survival rates. Adaptive immune cells, such as T cells, B cells, and macrophages, support an active response controlled by negative regulators, known as immune checkpoints (i.e., PDL1, CTLA4, and CD274), resulting in a strictly regulated antitumor response [29]. Although immune cells assist in the immune system’s defense against tumor cells, an inflammatory TME contributes to the tumor’s aggressiveness, affecting treatment and outcomes [50]. Notably, despite some areas of disagreement, many of the identified prognostic clusters shared common features in gene expression and mutations.

Several clinical factors have been reported to be predictive and prognostic factors for ICC, such as NLR, TBS, CA 19-9, and CEA, alone or in combination [39,51,52]. In this regard, it is essential to determine if the different molecular subtypes have unique clinical and pathological characteristics. Connecting molecular markers and clinical characteristics would facilitate patient classification, which is beneficial for personalized treatment development. This may be crucial for decision-making in centers that cannot routinely perform molecular profiling. In the current review, only a few studies thoroughly addressed the association between clinicopathological characteristics and molecular clusters [21,26,29]. After conducting a subgroup analysis, the reported clinicopathological features had a low correlation and significant heterogeneity between studies; however, these features were associated with survival and could be used as prognostic markers. Although there was evidence that several clinical factors can predict survival in the entire cohort, there were few noticeable differences when analyzed within prognostic molecular subgroups. This can be explained by the fact that the reviewed studies aimed to identify molecular markers relevant to prognosis rather than identify ICC subtypes based on clinicopathological characteristics in addition to molecular profile. As such, further and well-designed studies are needed to overcome the limitations of the current ICC classification systems, which are solely based on molecular characteristics. This is necessary in order to comprehensively define specific ICC subgroups with distinct prognoses.

It is essential to consider certain limitations when interpreting the results of this review. First, the sample size for multi-omics-derived subtypes of ICC was limited due to the rare incidence of the disease and the limited evidence in the current literature. The exclusion of studies analyzing data from public registries also limited the sample size. This was necessary to avoid duplicated cohorts. However, a strength of this study was the inclusion of a diverse global cohort with almost equal representation of Western and Eastern ethnicities (42.1% and 57.9%, respectively). Second, the studies utilized a variety of methodologies for molecular profiling and different and few clinicopathological characteristics were investigated, which may result in the possibility of bias. Third, the management of patients with ICC has changed over time due to improvements in the knowledge of the disease and advancements in surgical and oncological treatments. The current review includes patients who were treated between 1991 and 2021. As such, a majority of patients were treated prior to the publication of BILCAP randomized controlled trial for standard chemotherapy and further clinical trials for target therapy that have been changing the approach to adjuvant chemotherapy recently [53,54]. In addition, it is important to note that the included studies reported few data on the treatment characteristics. As such, we could not adjust the results of the review for the treatment characteristics due to the lack of data.

## 5. Conclusions

The current review is relevant because it can clarify the insufficient and contradictory evidence in the existing literature of molecular-based ICC subtype classification with prognostic value and correlation with clinical factors. Although some reported classifications share specific points, such as the association between molecular subtypes and long-term outcomes, a clear correlation between molecular and gene expression markers and clinical features is not evident due to the study design and the lack of available data. Only a few studies have examined the relationship between multi-omics characterization and clinical patient stratification. Overall, an expression profile of genes that promote inflammation, mutations in the KRAS gene, large duct type, an advanced tumor stage, and elevated levels of CEA and CA19-9 predict a poor prognosis. This is one of the initial efforts to connect molecular characteristics with clinical factors in order to identify clinical markers of ICC clusters with different prognoses, select patients who should undergo tumor molecular characterization and targeted therapies, and tailor patient management promptly.

## Figures and Tables

**Table 1:** Characteristics of the studies.

Study ID	Patients	Age	Country	Time Frame	Outcomes
	*n*	Mean (Range)			
Ahn 2019 [21]	30	65.0 (49.0–79.0)	Korea	2008–2013	OS, DFS
Andersen 2012 [22]	104	64.0	Europe, US, Australia	1991–2008	OS, DFS
Bao 2022 [25]	110	60.0 (54.0–66.0) ^a^	China	2014–2021	OS
Cho 2023 [24]	102	64.0 (38.0–91-0)	Korea	2001–2018	OS, DFS
Dong 2022 [26]	262	61.0	China	2014–2018	OS
Goeppert 2019 [28]	52	59.9	Europe	-	OS
Job 2020 [29]	78	62.0 (52.0–69.0) ^a^	France	2001–2014	OS
Oishi 2012 [27]	23	59.5	China, Japan	2002–2003; 2008–2010	OS
Sia 2013 [23]	149	64.0 (54.0–70.0) ^a^	Europe, US	1995–2007	OS, DFS

^a^ Median value (IQR). Abbreviations: IQR, interquartile range; US, United States; OS, overall survival; DFS, disease-free survival.

**Table 2:** Characteristics of the clusters.

Study ID	Clusters	Good Prognosis Cluster	Bad Prognosis Cluster	Classifier	Validation
	*n*	*n* of patients (%)	*n* of patients (%)		
Ahn 2019 [21]	2	12 (37.5)	18 (56.3)	Yes	Yes
Andersen 2012 [22]	2	51 (49.0)	53 (51.0)	Yes	Yes
Bao 2022 [25]	3	33 (30.0)	37 (33.6)	No	Yes
Cho 2023 [24]	3	35 (34.3)	25 (24.5)	Yes	Yes
Dong 2022 [26]	4	67 (25.6)	41 (15.6)	No	Yes
Goeppert 2019 [28]	4	14 (26.9)	13 (25.0)	No	No
Job 2020 [29]	4	8 (10.3)	14 (17.9)	Yes	Yes
Oishi 2012 [27]	2	15 (65.2)	8 (34.8)	Yes	Yes
Sia 2013 [23]	2	57 (38.3)	92 (61.7)	Yes	Yes

**Table 3:** Molecular and mutational patterns of clusters.

		Mutations						Gene Expression Signature *					Prognosis	
Study ID	Cluster	KRAS	TP53	IDH1	FGFR2	BRAF	ARID1A	Inflammation	Proliferation	Mesenchymal	Immune	Metabolic	Good	Bad
Ahn 2019 [21]	1			+	+							+	+	
	2	+	+					+	+		+			+
Andersen 2012 [22]	1										+		+	
	2	+				+			+	+				+
Bao 2022 [25]	1				+		+		+				+	
	2											+		
	3	+	+					+						+
Cho 2023 [24]	1			+						+		+	+	
	2													
	3	+	+								+			+
Dong 2022 [26]	1				+								+	
	2		+						+			+		
	3								+	+				
	4	+						+						+
Goeppert 2019 [28]	1												+	
	2			+										
	3													
	4													+
Job 2020 [29]	1		+					+			+		+	
	2													
	3			+						+				
	4	+							+	+				+
Oishi 2012 [27]	1												+	
	2									+				+
Sia 2013 [23]	1				+			+					+	
	2		+						+					+

* If the study did not clearly define the gene expression signature as inflammatory, proliferation, mesenchymal, immune, or metabolic, a consensus was reached among the investigators based on the molecular data provided by each study. A positive enrichment of the reported mutations or gene expression signature is indicated by the symbol “+”.

**Table 4:** Survival analysis.

Study ID	Patients	Clusters’ Median OS				Clusters’ 3-Yr OS			Clusters’ 5-Yr OS				
	*n*	Months				%			%				
		1	2	3	4	1	2	3	4	1	2	3	4
Ahn 2019 [21]	30	NA			25.3	70.7			25.8	70.7			7.6
Andersen 2012 [22]	104	NA			22.0	73.0			43.0	72.0			30.0
Bao 2022 [25]	110	NA	30.0		15.9	58.8	21.5		15.9				
Cho 2023 [24]	101	63.0	42.0		11.0	70.3	54.3		25.3	56.0	42.7		25.3
Dong 2022 [26]	214	NA	38.3	26.3	18.2	74.4	53.0	37.2	30.3	67.1	46.7	36.2	21.8
Goeppert 2019 [28]	49	NA	48.2	36.2	12.3	91.2	65.0	38.7	30.0	78.0	32.1	27.9	14.2
Job 2020 [29]	76	72.6	41.4	24.6	16.3	74.1	58.0	34.0	20.0	59.3	42.4	33.4	9.8
Oishi 2012 [27]	23	NA			28.9	63.1			0				
Sia 2013 [23]	119 ^a^	47.7			24.4	56.6			41.4	39.8			28.3

NA: not available. ^a^ 119/149 patients had data on survival.

**Table 5:** Clinicopathological characteristics of good and bad prognosis clusters.

	Cluster	Ahn 2019 [21]	Andersen 2012 [22]	Bao 2022 [25]	Cho 2023 [24]	Dong 2022 [26]	Goeppert 2019 [28]	Job 2020 [29]	Oishi 2012 [27]	Sia 2013 [23]
CEA < 5 ng/mL, *n* (%)	Good	11 (91.7) *				61 (91.0) *				
	Bad	9 (50.0) *				27 (65.8) *				
CEA > 5 ng/mL, *n* (%)	Good	0 *				6 (8.9) *				
	Bad	7 (38.9) *				14 (34.1) *				
HBV, *n* (%)	Good	4 (33.3) *		26 (78.8)			1 (6.7)	0		6 (13.0)
	Bad	0 *		30 (81.1)			0	1 (7.1)		5 (7.0)
Tumor size (cm)	Good	6.3 ± 6.0 ^a^	6.7 ± 0.43 ^a^							6 (4–8.4) ^b^
	Bad	6.0 ± 2.9 ^a^	5.1 ± 0.43 ^a^							7 (4.0–10.0) ^b^
Nodal metastases, *n* (%)	Good	2 (16.7)				4 (6.0) *				
	Bad	5 (27.8)				10 (24.4) *				
TNM stage, *n* (%)										
I	Good	7 (58.3)		I + II 24.0 (72.7)		34 (50.7) *	8 (53.3)	3 (37.5)		I + II 37 (78.0)
	Bad	8 (44.4)		I + II 20.0 (54.1)		6 (14.6) *	4 (28.6)	0		I + II 57 (79.0)
II	Good	3 (25.0)				20 (29.8) *	5 (33.3)	1 (12.5)		
	Bad	3 (16.7)				18 (43.9) *	8 (57.1)	8 (57.1)		
III	Good	0		III + IV 9.0 (27.3)		12 (17.9) *	2 (13.3)	0		III + IV 9 (19.0)
	Bad	1 (5.6)		III + IV 17.0 (46.0)		12 (29.3) *	2 (14.3)	2 (14.3)		III + IV 15 (21.0)
IV	Good	2 (16.6)				1 (1.5) *	0	3 (37.5)		
	Bad	6 (33.3)				5.0 (12.2) *	0	2 (14.3)		
VI, *n* (%)	Good	5 (41.7)				23 (34.3)		3 (37.5)		4 (9.0)
	Bad	7 (38.8)				20 (42.5)		6 (42.5)		12 (17.0)
Histological type *n* (%)										
Large duct	Good	2 (6.1) *	9 (25.0)				5 (33.3)			
	Bad	13 (72.2) *	27 (75.0)				5 (35.7)			
Small duct	Good	10 (83.3) *	42 (61.8)				10 (66.7)			
	Bad	5 (27.8) *	26 (38.2)				9 (64.3)			
Grade, *n* (%)										
G1	Good							3 (37.5)		
	Bad							3 (37.5)		
G2	Good							3 (37.5)		
	Bad							9 (64.3)		
G3	Good							2 (25.0)		
	Bad							2 (14.3)		
PNI, *n* (%)	Good		14 (29.8) *			4 (5.9) *		2 (25.0)		2 (4.0) *
	Bad		28.0 (62.2) *			13.0 (24.4) *		4 (28.6)		18 (25.0) *

Abbreviations: PNI, perineural invasion; VI, vascular invasion; LC, liver cirrhosis; HBV, hepatitis B virus. * *p* < 0.05 (good vs. bad prognosis clusters). ^a^ Mean ± standard deviation. ^b^ Median (interquartile range).

## Data Availability

No new data were created or analyzed in this study. Data sharing is not applicable to this article.

## Supplementary Materials

The following supporting information can be downloaded at: https://www.mdpi.com/article/10.3390/cancers16142596/s1, Figure S1. Forest plot for overall survival based on multivariable analysis. Figure S2. Forest plot for risk factors based on univariable analysis: (A) TNM stage; (B) Perineural invasion; (C) Vascular invasion; (D) Funnel plot for publication bias. Table S1. Newcastle–Ottawa Scale for Quality Assessment. Table S2. Multi-omics sequencing and clustering method. Table S3. Genetic alterations. Table S4. Hazard ratios for survival. Table S5. Clinicopathological characteristics. Table S6. Univariable and multivariable analyses for clinicopathological characteristics relative to overall survival.
