# Supplementary material for: Multi-Omics Classification of Intrahepatic Cholangiocarcinoma: A Systematic Review and Meta-Analysis
Source: Cancers (Basel). 2024 Jul 20;16(14):2596. doi: 10.3390/cancers16142596 (PMC11275091; doi:10.3390/cancers16142596)

Supplementary Material

Multi-omics Classification of Intrahepatic Cholangiocarcinoma: A Systematic Review and Meta-analysis

Laura Alaimo [1], Sara Boggio [1], Giovanni Catalano [1], Giuseppe Calderone [1], Edoardo Poletto [1], Mario De Bellis [1], Tommaso Campagnaro [1], Corrado Pedrazzani [1], Simone Conci [1], Andrea Ruzzenente [1*]

[1] Department of Surgery, Dentistry, Gynecology, and Pediatrics, Division of General and Hepato-Biliary Surgery, University of Verona, University Hospital G.B. Rossi, Verona, Italy

[*] Correspondence: A.R. andrea.ruzzenente@univr.it General and Hepato-Biliary Surgery; Department of Surgery, Dentistry, Gynecology, and Pediatrics; University of Verona; P.le L.A. Scuro 10, 37134 Verona, Italy; Tel. +39 045 8124411

+Laura Alaimo and Sara Boggio contributed equally to this work.

Supplementary Tables

Supplementary Table S1. Newcastle Ottawa Scale for Quality Assessment.

	**Patient Selection**		**Comparability**		**Outcome**				**Total**
**Study ID**	**Selection**	**Sample size**	**Markers**	**Clustering**	**Gene signatures**	**Survival**	**Clinicopath ological**	**Validation**	
Ahn 2019[21]	1	1	1	1	1	1	1	1	8/8
Andersen 2012[25]	1	1	1	1	1	1	1	1	8/8
Bao 2022[22]	1	1	1	1	0	0	1	1	6/8
Cho 2023[52]	1	1	1	1	1	1	0	1	7/8
Dong 2022[23]	1	1	1	1	0	1	1	1	7/8
Goeppert 2019[26]	1	1	1	0	0	1	1	0	5/8
Job 2020[27]	1	1	1	1	1	1	1	1	8/8
Oishi 2012[24]	1	1	0	1	1	0	0	1	5/8
Sia 2013[28]	1	1	1	1	1	1	1	1	8/8

Supplementary Table S2. Multi-omics sequencing and clustering method.

Study ID	Sequencing platform	Data	Clustering method
Ahn 2019[21]	RNAseq (Illumina)	Transcriptomic	Unsupervised hierarchical
Andersen 2012[25]	Microarray (Illumina)	Transcriptomic	Hierarchical
Bao 2022[22]	Tandem-mass tag (ThermoFisher)	Proteomic	Consensus clustering (hierarchical)
Cho 2023 [52]	Tandem-mass tag (ThermoFisher)	Proteomic	Unsupervised hierarchical
Dong 2022[23]	Tandem-mass tag (ThermoFisher)	Proteomic	Unsupervised consensus (hierarchical)
Goeppert 2019[26]	WES (Illumina), Methylome BeadChip (Illumina)	CNAs, Genomic, Methylomic	Integrative
Job 2020[27]	Microarray (Affymetrix)	Transcriptomic	Hierarchical
Oishi 2012[24]	Microarray (Affymetrix)	Transcriptomic	Unsupervised hierarchical
Sia 2013[28]	Microarray (Illumina)	Transcriptomic	Unsupervised hierarchical

Supplementary Table S3. Genetic alterations.

Study ID	Mutations							
	**KRAS**	**TP53**	**IDH1**	**FGFR2**	**BRAF**	**ARID1A**	**BAP1**	**ERBB2**
Ahn 2019[21]	X	X	X	X				
Andersen 2012[25]	X				X			
Bao 2022[22]	X	X			X	X		
Cho 2023[52]								
Dong 2022[23]	X	X	X	X		X	X	
Goeppert 2019[26]			X		X		X	X
Job 2020[27]			X					
Oishi 2012[24]								
Sia 2013[28]	X				X			

Supplementary Table S4. Hazard ratios for survival.

Study ID	Reference	Univariable analysis		Multivariable analysis	
		HR (95%CI)	p value	HR (95%CI)	p-value
Ahn 2019[21]	Good prognosis cluster	2.95 (1.54-5.67)	0.001	7.45 (1.24–1.36)	0.020
Andersen 2012[25]	Good prognosis cluster	2.27 (1.20-4.10) *	0.007	4.76 (1.00-23.00)	0.051
Bao 2022[22]	Good prognosis cluster	2.62 (1.45-4.70)	0.001		
Cho 2023[52]	Good prognosis cluster	1.34 (0.71-2.52)	0.004		
Dong 2022[23]	Good prognosis cluster	2.63 (1.42-4.87)	<0.001	1.27 (1.02-1.59)	0.033
Goeppert 2019[26]	Good prognosis cluster	1.91 (0.49-7.38)	0.005		
Job 2020[27]	Good prognosis cluster	2.60 (1.30-5.10)	0.005	2.3 (0.63-8.5)	0.210
Oishi 2012[24]	Good prognosis cluster	1.72 (1.00-3.03) *	0.056		
Sia 2013[28]	Good prognosis cluster	1.59 (1.00-2.51)	0.048		

* Data from Job et al.[25].

Supplementary Table S5. Clinicopathological characteristics.

Study ID	Patients	LC	HBV	CEA, μg/L	CA19-9, U/mL	Tumor size, cm	TNM stage				PNI	VI
							I	II	III	IV		
	n	n (%)	n (%)	Mean (Range)	Mean (Range)	Mean (Range)	n (%)				n (%)	n (%)
Ahn 2019[21]	30	-	4(12.0)	-	-	6.5(6.0-6.3)	15(50.0)	6(20.0)	1(3.3)	8(26.6)	-	12(40.0)
Andersen 2012[25]	104	-	-	-	-	-	-	-	-	-	80(76.9)	-
Bao 2022[22]	110	21(19.1)	93(84.5)	-	-	-	50(45.2)	18(16.3)	42(38.2)	-	-	-
Cho 2023[52]	102	15(14.7)	19(18.6)	-	-	-	-	-	-	-	-	45(44.1)
Dong 2022[23]	262	24(9.1)	79(30.0)	2.9(0.5-911.3)	83.3(0.6-10,000)	5.5(1.3-15.0)	84(32.0)	81(31.0)	84(32.0)	13(5.0)	54(21.0)	107(41.0)
Goeppert 2019[26]	52	-	3(5.8)	2.7(0.4-301.8)	46.2(0.6-10,000)	7.2	16(30.8)	28(53.8)	7(13.4)	1(1.9)	-	-
Job 2020[27]	78	12(5.4)	3(4.6)	-	73.0(18.0-527.0)[a]	-	-	-	-	-	22(28.2)	51(65.4)
Oishi 2012[24]	23	8(33.3)	8(33.3)	-	-	-	-	-	-	-	-	-
Sia 2013[28]	119[b]	20(17.0)	11(9.0)	-	-	6.0(4.0-9.0)[a]	-	-	-	-	17(17.0)	15(13.0)

Abbreviations: PNI, perineural invasion; VI, vascular invasion; LC, liver cirrhosis; HBV, hepatitis B virus; CEA, carcinoembryonic antigen; CA19-9, carbohydrate antigen 19-9. [a] Median (IQR). [b] 119/149 patients had data on survival. The variables included had less than 10% of missing values

Supplementary Table S6. Univariable and multivariable analyses for clinicopathological characteristics relative to the overall survival.

Study ID	Variables	Reference	Univariable Analysis		Multivariable Analysis	
			HR (95% CI)	p-value	HR (95%CI)	p-value
	CEA	≤ 5 ng/mL				
Ahn 2019[21]			2.92 (1.20-7.11)	0.02		
Dong 2022[23]				< 0.001	2.64 (1.65-4.23)	< 0.001
	CA19-9	≤ 37 U/mL				
Ahn 2019[21]			1.55 (0.74-3.24)	0.25		
Dong 2022[23]				0.01		
	Tumor size	≤ 6 cm				
Sia 2013[28]			1.69 (1.08-2.63)	0.02	1.92 (1.13-3.27)	0.02
Dong 2022[23]				0.01		
	TNM stage	I-II				
Ahn 2019[21]			3.19 (1.67-6.12)	< 0.001	9.76 (2.45–38.91)	0.001
Dong 2022[23]				< 0.001	2.03 (1.03-4.01)	0.04
Sia 2013[28]			2.50 (1.49-4.18)	< 0.001		
	PNI	No				
Andersen 2012[25]				< 0.001		
Dong 2022[23]				< 0.001	1.69 (1.01-2.83)	0.04
Job 2020[27]			1.45 (0.78-2.70)	0.20		
Sia 2013[28]			2.41 (1.40-4.15)	<0.01		
	VI	No				
Ahn 2019[21]			1.89 (0.92–3.90)	0.08		
Dong 2022[23]				<0.001		
Job 2020[27]			1.44 (0.80-2.60)	0.2		
Sia 2013[28]			2.49 (1.38-4.49)	<0.01	3.17 (1.68–5.95)	0.04

Abbreviations: PNI, perineural invasion; VI, vascular invasion.

Supplementary Figures

Supplementary Figure S1. Forest plot for overall survival based on multivariable analysis.

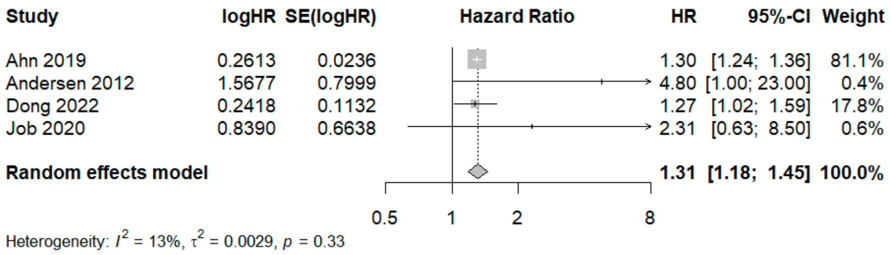

Supplementary Figure S2. Forest plot for risk factors based on univariable analysis: (**A**) TNM stage; (**B**) Perineural invasion; (**C**) Vascular invasion; (**D**) Funnel plot for publication bias.

A

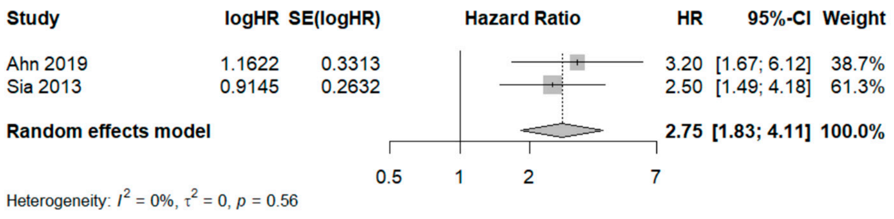

B

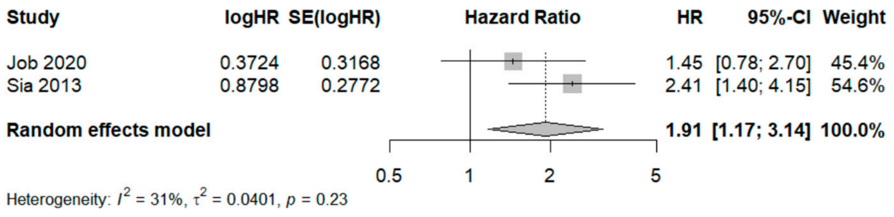

C

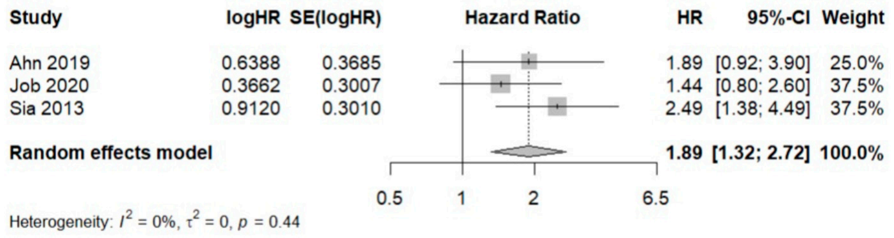

D

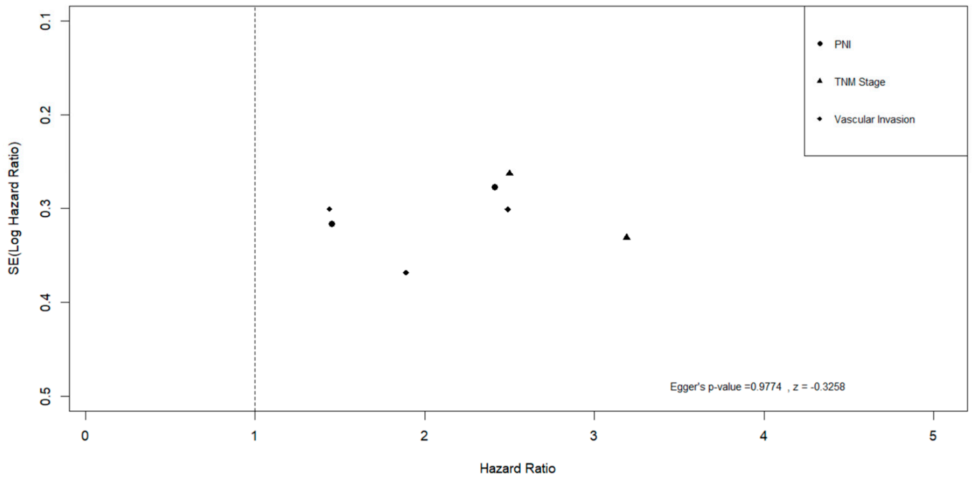

Supplement: Supplementary file 1 [file cancers-16-02596-s001.zip › cancers-3063449-supplementary.pdf]
